# Lipid Tail Protrusion in Simulations Predicts Fusogenic Activity of Influenza Fusion Peptide Mutants and Conformational Models

**DOI:** 10.1371/journal.pcbi.1002950

**Published:** 2013-03-07

**Authors:** Per Larsson, Peter M. Kasson

**Affiliations:** Departments of Molecular Physiology and Biological Physics and of Biomedical Engineering, University of Virginia, Charlottesville, Virginia, United States of America; University of Illinois, United States of America

## Abstract

Fusion peptides from influenza hemagglutinin act on membranes to promote membrane fusion, but the mechanism by which they do so remains unknown. Recent theoretical work has suggested that contact of protruding lipid tails may be an important feature of the transition state for membrane fusion. If this is so, then influenza fusion peptides would be expected to promote tail protrusion in proportion to the ability of the corresponding full-length hemagglutinin to drive lipid mixing in fusion assays. We have performed molecular dynamics simulations of influenza fusion peptides in lipid bilayers, comparing the X-31 influenza strain against a series of N-terminal mutants. As hypothesized, the probability of lipid tail protrusion correlates well with the lipid mixing rate induced by each mutant. This supports the conclusion that tail protrusion is important to the transition state for fusion. Furthermore, it suggests that tail protrusion can be used to examine how fusion peptides might interact with membranes to promote fusion. Previous models for native influenza fusion peptide structure in membranes include a kinked helix, a straight helix, and a helical hairpin. Our simulations visit each of these conformations. Thus, the free energy differences between each are likely low enough that specifics of the membrane environment and peptide construct may be sufficient to modulate the equilibrium between them. However, the kinked helix promotes lipid tail protrusion in our simulations much more strongly than the other two structures. We therefore predict that the kinked helix is the most fusogenic of these three conformations.

## Author Summary

Membrane fusion is a common process critical to both cellular function and infection by enveloped viruses. Influenza is a particularly useful model system for studying fusion because the fusion reaction is accomplished by a single protein, hemagglutinin. Furthermore, mutations to the membrane-inserted portion of hemagglutinin have been identified that do not detectably alter the rest of the protein but can either arrest fusion halfway or block it entirely. For influenza at least, it seems that the membrane-inserted hemagglutinin peptide plays a critical role in promoting fusion, perhaps by increasing the local disorder of lipid bilayers. However, we lack a mechanistic understanding sufficient to predict the activity of fusion peptide mutants from their sequence. Here, we have used lipid tail protrusion as a way to measure how much fusion peptides disorder their surrounding bilayer; we see a strong relationship between lipid tail protrusion and the ability of fusion peptide mutants to promote lipid mixing between membranes. Our simulations also predict that this lipid tail protrusion is much more common when the peptides adopt a kinked helix structure than when they are straight or hairpin-like. We therefore hypothesize that, while all three types of structure likely undergo conformational exchange, the kinked helix structure is most active in promoting fusion.

## Introduction

Membrane fusion is critical to eukaryotic cellular function and also provides the mode of entry for enveloped viruses such as influenza and HIV. Influenza viral entry is mediated by the hemagglutinin protein. As part of this process, short fusion peptides are inserted into the host membrane and act to promote fusion. Influenza is a distinctive system for studying fusion because hemagglutinin mutants have been generated that can insert fusion peptides and pull viral and target membranes together but not complete the fusion process [1]–[5]. This can be accomplished via mutations in the fusion peptide region or deletions in the transmembrane domain. These mutagenesis results suggest a specific role for fusion peptide-membrane interactions in promoting influenza membrane fusion. The mechanism by which fusion peptides act on membranes remains unknown, but some possibilities that have been previously suggested include inducing membrane curvature, altering local membrane composition, and inducing local disorder in membrane lipids [6]–[9].

**Figure 1:**
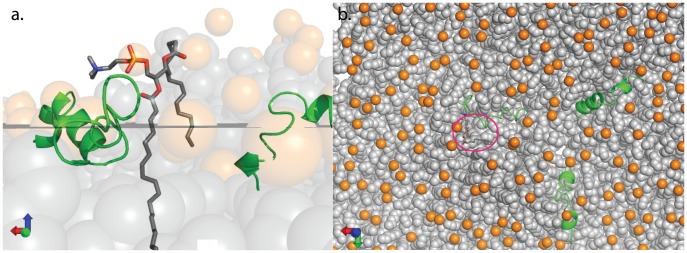
Lipid tail protrusion. This simulation snapshot is shown in side (a) and top (b) views and highlights one lipid, rendered in sticks, with an acyl tail protruding into the polar layer. One acyl tail of this lipid is bulging into the polar layer, extending >1 Å past its phosphorus atom and constituting what we classify as a protrusion event. The lipid is located near a fusion peptide (green). Other lipid tails are shown in gray spheres and other phosphates in orange spheres. A thin gray plane shows the average phosphorus position in the upper leaflet [46]. The fusion peptides are membrane-inserted but sit in a local undulation of the bilayer, hence the local abundance of lipids above the average *z*-position.

Because of the dynamic and heterogeneous nature of membrane assemblies and the transience of fusion intermediates, molecular dynamics simulations have been used to generate and examine physical hypotheses for fusion mechanisms. These simulations have suggested that hydrophobic tail protrusion into the polar layer between two apposed bilayers (Fig. 1) may be an important feature and indeed a transition state for fusion stalk formation [10]–[13]. We have previously shown that influenza fusion peptides can promote lipid tail protrusion in simulations without loss of overall lamellar structure [10]. However, these predictions and their consequences for influenza fusion are difficult to test spectroscopically.

Several structural models exist for native hemagglutinin fusion peptides in membranes, obtained via different approaches and under different conditions. NMR experiments in micelles and EPR experiments in bilayers have provided a kinked helix model for the structure of the fusion peptide of X-31 (H3) hemagglutinin in bilayers. This model is consistent with additional infrared and circular dichroism spectroscopic data [6], [14], [15]. Solid-state NMR experiments in bilayers have yielded structures that are grossly similar but have a slightly more pronounced kink [16]. More recently, NMR studies in micelles using a longer construct from A/swine/Scotland/410440/94 (H1) hemagglutinin have yielded a helical hairpin structure [17], [18]. Finally, simulation studies have also suggested that a relatively flat helical model may be appropriate [19], [20], though other simulations have yielded a kinked helix [21], [22] or rapid exchange between the two [19], [23]. Additional NMR structural data are available for a series of fusion peptide mutants [24], including the N-terminal glycine mutants discussed below.

Here, we wish to better understand how influenza fusion peptides can drive fusion. We first report an indirect test of tail protrusion as a surrogate outcome for catalysis of fusion stalk formation in influenza membrane fusion. This is accomplished by simulating mutant fusion peptides with known experimental phenotypes and comparing lipid protrusion in these simulations to experimental fusogenic activity. We then use tail protrusion as a means to examine how different fusion peptide conformations may contribute to fusogenic activity. We find that all three major postulated conformations are accessible in our simulations. Since the precise conformational equilibria are likely controlled by membrane environment, peptide sequence, and sample preparation, this study does not address the question of which peptide conformation predominates under the membrane conditions of fusion. Indeed, both bilayer lipid composition and choice of detergent for membrane protein structure determination can substantially affect protein structure [25], [26]. Instead, we use simulations to analyze how each of these conformations may contribute to lipid protrusion and fusion activity.

## Results

**Figure 2:**
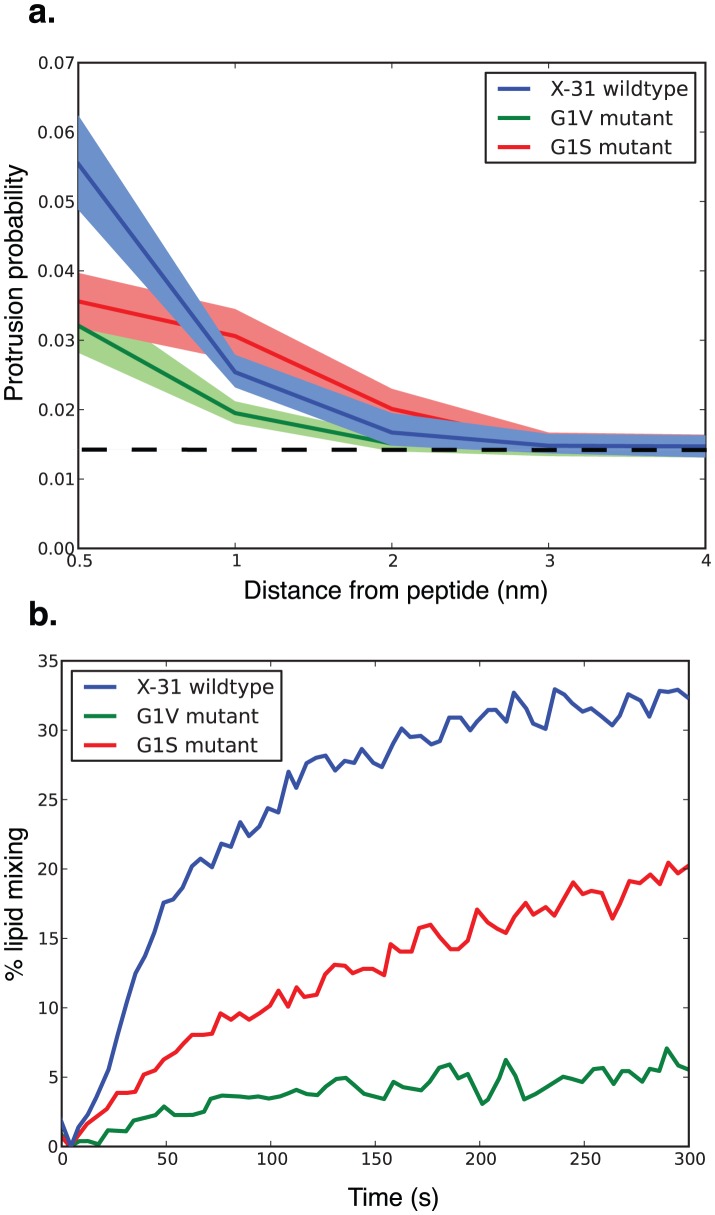
Lipid tail protrusion is increased close to fusion-active peptides. Protrusion probability is plotted in (a) as a function of distance from the nearest peptide for the full set of 600 trajectories, and compared with fusion kinetics (b) measured via lipid mixing for transfectants expressing each mutation replotted from Qiao et al. [2]. The protrusion probability is greatest for fusion peptides with an N-terminal glycine (X-31 wild-type). Protrusion is approximately half as likely near G1V mutant peptides (p<0.01, Kolmogorov-Smirnov test). The G1S peptide shows an intermediate behavior. Shaded areas denote 95% confidence intervals calculated using bootstrap resampling.

We performed molecular dynamics simulation of influenza fusion peptides and mutants in lipid bilayers and evaluated the resulting trajectories for lipid tail protrusion close to the peptides. A protrusion event was defined as any carbon in the lipid tail protruding more than 0.1 nm beyond the phosphorus atom of that lipid (Fig. 1). Lipid protrusion probabilities are plotted in Fig. 2a as a function of distance from the nearest fusion peptide. As hypothesized, lipids near the G1V mutant peptide showed significantly less protrusion (p<0.01, Kolmogorov-Smirnov test) than lipids near the wild-type X-31 peptide, displaying only a minimal increase over baseline. Lipids near the G1S mutant peptide showed protrusion intermediate between the wild-type and G1V peptides (p<0.01 via Kolmogorov-Smirnov with Bonferroni correction), consistent with the experimental observation that G1S hemagglutinin catalyzes lipid mixing between fusion partners (a measurement of early fusion intermediate formation) but less efficiently than wild-type (Fig. 2b). Further testing of additional mutants will be helpful, but our simulation results to date support lipid tail protrusion as a predictive measure of fusogenic activity.

Protrusion is a not-infrequent event in pure lipid bilayers, although it appears to be specifically enhanced by fusogenic peptides. Based on the probability of protrusion in simulations of a pure 1-palmitoyl 2-oleoyl phosphatidylcholine (POPC) bilayer, we estimate the free energy cost for any acyl chain carbon of a lipid to protrude at least 0.1 nm at approximately 0.5 kT; this increases to 2.2 kT if a protrusion threshold of 0.2 nm is used. We have previously tested protrusion in the POPC bilayer surrounding an ion channel, and no significant increase was observed over a protein-free bilayer, suggesting that protrusion is not a general phenomenon near inserted proteins but likely more specific to membrane-disordering and perhaps fusion peptides [10]. Furthermore, at the 1∶167 peptide∶lipid ratio used, enhancement of protrusion was a local effect: at long distance from the peptides, protrusion probabilities are identical within error to protein-free bilayers. Calculated S_CD_ order parameters for peptide-free and peptide-inserted bilayers indicate that the membranes remain lamellar in the presence of peptides and are not grossly disordered (Fig. S1).

To test for direct peptide-lipid interactions that might result in protrusion, we examined interaction energies between fusion peptides and each lipid in their immediate surroundings. These energies were computed at 1 ns intervals for all lipids within 12 Å of a peptide, using the AMBER03 force field, over the course of a 100-ns simulation of the X-31 fusion peptide and one of the G1V mutant. Lipids with a protruding acyl tail did not show a significant difference in interaction energy with the protein compared to lipids that did not protrude (Fig. S5). This suggests that the majority of lipids with protruding acyl tails are not participating in strong interactions with the peptide that would drive such behavior.

**Figure 3:**
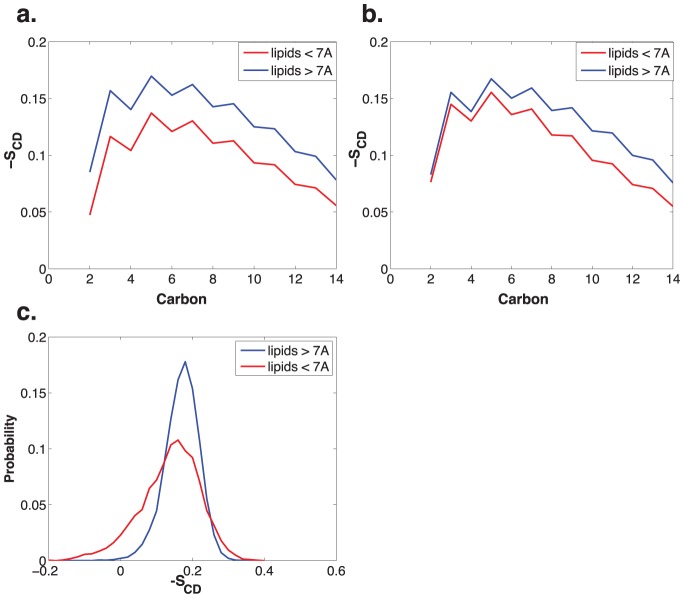
Fusion-active peptides induce decreased acyl tail order in nearby lipids. Calculated carbon-deuterium order parameters are plotted for sn-1 acyl tails in (a) for lipid bilayers containing the X-31 fusion peptide and in (b) for bilayers containing the G1V mutant. X-31 peptides induce a significant disordering of lipid tails within 7 Å compared to tails in the same leaflet but >7 Å. The G1V peptide induces a significantly smaller effect, perhaps related to its lesser promotion of lipid protrusion. Values in (a–b) are averaged over lipids. Plotted in (c) is the distribution of carbon-deuterium order values (sign reversed from panels a–b) for carbon 4 of sn-1 tails in the X-31 simulations. Values show a significant deviation from normality (p<0.001, Shapiro-Wilk test) with substantial “tail” population of more disordered lipids close to the peptide. The Pearson correlation coefficient between calculated SCD parameters in the X-31 simulations and experimentally measured parameters [47] is 0.92.

Fusion peptides appear to promote acyl tail protrusion via a local change in bilayer order rather than either a global disordering of the bilayer or highly specific intermolecular interactions. While average S_CD_ order parameters were displayed only a slight shift between peptide-containing and peptide-free bilayers, there was a significant change in order parameters of lipids very close to the peptide (Fig. 3). To distinguish generic interfacial effects from those specific to active fusion peptides, we compared lipid order parameters in the X-31 wild type peptide simulations and those of the fusion-null G1V mutant. Lipids closest to the X-31 peptide (within 7 Å of the nearest peptide atom, approximately the first lipid shell) showed a significant reduction in order parameters, while lipids in the same leaflet but farther than 7 Å did not (Fig. 3a; p<0.001, Kolmogorov-Smirnov test for each carbon C3–C14). Lipids close to the G1V mutant peptide displayed a significantly smaller effect (p<0.001, Kolmogorov-Smirnov; Fig. 3b). This finding provides additional evidence of a highly localized disordering effect specifically in the presence of active fusion peptides.

Fusion-active peptides thus appear to induce substantial disorder in a small fraction of nearby lipids. One possible mechanism for this is partial insertion of the peptide into the bilayer outer leaflet, displacing volume in the hydrocarbon region and leaving a localized pressure imbalance in the polar region. In our simulations, we occasionally observe a lipid “straddling” the partially inserted fusion peptide helix (Fig. S4), with one acyl tail on each side of the helix. Such straddling conformations have been previously observed in crystallographic structures of aquaporins [27]. In our simulations, straddling lipids did not always protrude past the phosphate group, but when they did, the protrusion time appears longer. The average protrusion probability for lipids straddling a kinked helix conformation was significantly higher than distance-matched non-straddling “control” lipids (p<0.01, Kolmogorov-Smirnov test). Straddle-related acyl chain protrusion does not account for the full difference between fusion-active and fusion-null peptides. However, it may be an example of a more general phenomenon for how partial insertion of a kinked fusion peptide helix leads to lipid tail disordering and protrusion.

We also performed simulations to examine the fusogenic activity of different structural models for the influenza fusion peptide. In our simulations of the X-31 fusion peptide, the peptide sampled kinked-helix conformations, flat helical conformations, and helical hairpin conformations, coming within 1.2 Å C-alpha RMSD of NMR structural models for each of these (see Text S1 for details). We therefore performed additional simulations where the same X-31 fusion peptide was restrained to each of these conformations using an elastic network model. Six restrained simulations of up to 200 ns each were run per structural model. We measured lipid tail protrusion in these simulations as a surrogate for fusogenic activity. The constructs used for different NMR structural studies varied in length; all simulations reported here use residues 1–20 of influenza hemagglutinin.

**Figure 4:**
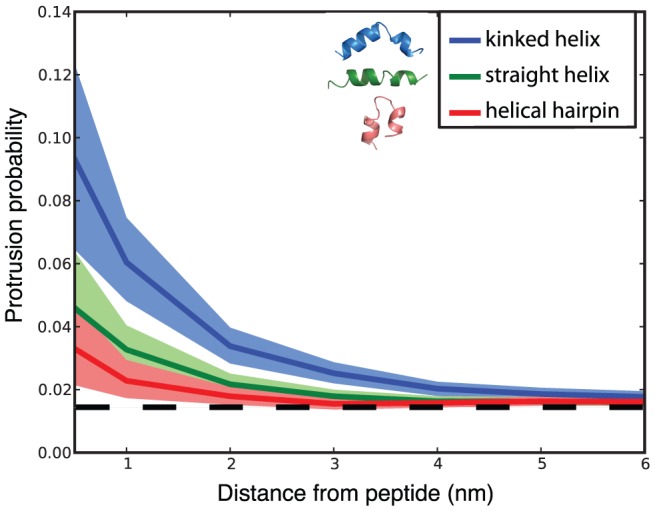
Kinked-helix conformations are most efficient at inducing lipid protrusion. Fusion peptide conformations were restrained to one of 3 models: a kinked helix (blue), a straight helix (green), or a helical hairpin (red). Lipid tail protrusion probability is plotted as a function of distance from peptide in the resulting simulations. Confidence intervals were calculated at 95% via bootstrap resampling. All simulations used residues 1–20 of the X-31 peptide sequence, restrained to each conformational model in turn.

The kinked helix produced by far the greatest probability of lipid protrusion (Fig. 4), significantly more than either the flat helix or hairpin simulations (p<0.01, Kolmorogov-Smirnov test). The increase in tail protrusion in our simulations takes the form of more frequent protrusions rather than longer persistence of each protrusion (Fig. S2). These results suggest that among the models tested here, whatever the equilibrium probability of each conformation at the conditions of fusion, the kinked helix has the greatest fusogenic activity.

**Figure 5:**
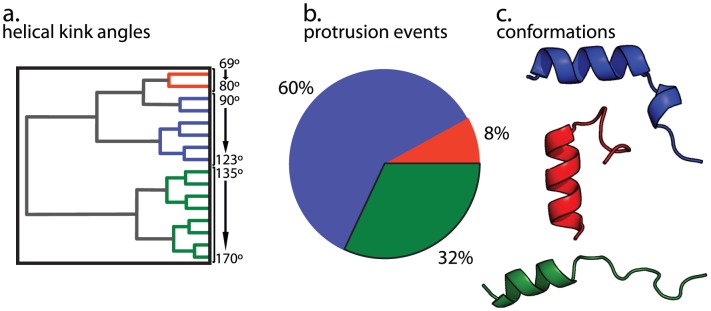
Protrusion events mapped to fusion peptide conformations for the unrestrained X-31 peptide simulation. For each protrusion event, the nearest fusion peptide was selected, and this collection of conformations was clustered by kink angle (a). Peptides were classified as kinked helix (blue), which account for 60% of protrusion events (b), hairpin-like (red), which account for 8% of protrusion events, and relatively flat (green), which account for 32% of protrusion events. Representative structures are rendered in panel (c).

**Figure 6:**
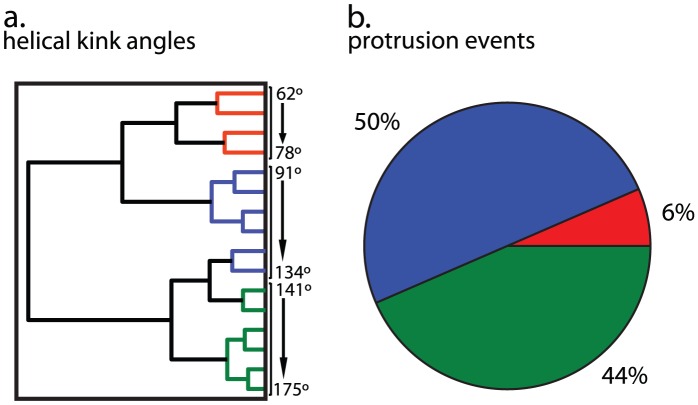
Protrusion events mapped to fusion peptide conformation for the unrestrained G1V-mutant simulation. Clustering (a), mapping (b) and classification was performed as in Fig. 5. Similar to the X-31 peptide (Fig. 5), the majority of protrusion events occur near a kinked helix (50%) followed by 44% close to more shallow kink-angles, and 6% around hairpin-like structures. The lower overall likelihood of a kinked helix conformation for the G1V mutant peptide may explain the lower protrusion probabilities.

As an additional test of the effect of fusion peptide conformation on tail protrusion, we analyzed our unrestrained simulations of the X-31 fusion peptide and asked what conformation the peptide adopted at the start of each protrusion event. 60% of all protrusion events originated from a kinked helix conformation (Fig. 5), 32% from a flat, predominantly helical conformation, and 8% from a helical hairpin conformation. Mean helical kink angles for each of these conformational groups were 110° for the kinked helices, 154° for the flat helices, and 74° for the hairpin-like structures (see Text S1). Similarly, the protrusion events that did occur in simulations of the G1V mutant peptide were most likely to occur when the peptide adopted a kinked-helix conformation (Fig. 6), which happened much less often than for the X-31 peptide. This assessment of which conformations are associated with the most protrusion events is of course biased by the fraction of time the simulations spent in each conformation. Nonetheless, we obtain the same results from two orthogonal approaches: the restrained simulations where we pre-suppose a set of structural models and equalize the sampling of each model and the unrestrained simulations where we have uneven sampling but discover protrusion-associated conformations in an unbiased fashion. Both suggest that a kinked helix is the most fusion-active conformation among those visited by our simulations.

## Discussion

Influenza fusion peptides clearly act to promote membrane fusion, but the mechanism of their action has been extremely difficult to probe. We have demonstrated that lipid protrusion probability near a mutant fusion peptide correlates with the ability of that hemagglutinin mutant to drive lipid mixing in cell transfectants and indeed with lipid mixing rates. This correlation supports the hypothesis that the transition state for stalk formation involves lipid tail protrusion and suggests that tail protrusion may be an effective surrogate outcome in evaluating fusogenic activity of peptides. Since our simulations of the X-31 fusion peptide sample conformations close to the kinked helix, flat helix, and helical hairpin structural models that have been previously proposed for influenza fusion peptide structure, we have used lipid tail protrusion to probe the fusogenicity of each conformation. Our results suggest that 1) the free-energy differences between these three states may be low enough that lipid environment and peptide sequence may have a substantial effect on the predominant population and 2) the kinked-helix state is significantly more fusogenic than the flat helix or helical hairpin.

Lipid tail exposure to the water layer, as would occur with tail protrusion, has been observed experimentally via NMR in POPC vesicles [28] as well as DPPC bilayers [29] and via neutron diffraction in DOPC bilayers [30]. In the NMR experiments, these are detected as Nuclear Overhauser Effect cross-relaxation between acyl-chain methyl and choline methyl groups. An increase in lipid tail protrusion due to fusion peptide activity should be detectable via such methods. However, two factors complicate the experiment. The first is that our simulations predict the effect on tail protrusion to be most profound in the upper region of the acyl chain, so a difference in tail exposure might be suboptimal probe for fusion peptide activity. Second, detecting an increase in average tail exposure over the entire sample might require a higher peptide∶lipid ratio than is physiological, since we predict tail protrusion to be a localized effect. If these two challenges can be overcome, these spectroscopic experiments would provide a good means to test our hypotheses.

Amphipathic helices have been observed to be membrane-active in a wide variety of physiological contexts. For influenza, it appears that the precise conformation of the helical residues has an effect on local membrane properties and may control fusion activity. Here, we have explored the relationship of fusion activity to leading structural models for influenza hemagglutinin. The physical determinants of fusion activity in systems like parainfluenza, where the prevailing model is that of a transmembrane helix for the fusion peptide [31] will be an interesting subject for future investigations. Another challenge for the future is to integrate simulation with spectroscopic data to examine how different membrane environments alter the conformational equilibria of fusion peptides and how this may act to regulate the efficiency of viral entry in cellular systems.

## Methods

To evaluate the relationship between lipid protrusion and fusogenic activity, we simulated a set of hemagglutinin mutants for which extensive structural and fusion activity data are available. The N-terminal glycine of HA2 is heavily conserved—among deposited human influenza hemagglutinin sequences [32], >99.9% have glycine in this position. Cells transfected with G1S hemagglutinin display a terminal hemi-fused phenotype and reduced lipid-mixing rates compared to wild-type X-31, and the G1V mutation blocks lipid mixing altogether [2]. This series of mutations thus provides a sensitive test of our protrusion hypothesis: the G1S mutant displays a block after stalk formation and would be expected to promote tail protrusion, while the fusion-null G1V mutant would not.

We therefore simulated three copies of each fusion peptide in a POPC bilayer at a 1∶167 peptide∶lipid ratio. All simulations were started from the kinked helix structure (PDB code 1IBN) docked into the bilayer according to EPR data [33] with appropriate mutations; the structural differences among mutants detected via NMR were not assumed. Lipids strongly overlapping the inserted fusion peptide were removed (approximately 4 per peptide); the criteria used for lipid deletion were phosphate groups within 7 Å of the peptide as previously reported [33]. Any protrusion propensity resulting from such a pressure imbalance would be a factor 2 lower than what we observe (Fig. S3).

Three copies of the fusion peptide and approximately 500 POPC molecules were solvated with TIP3P water in a box with sides 13 nm and height 6.5 nm in length. The AMBER03 force field [34] was used for protein parameters in conjunction with the Berger lipid parameters [35] as previously described [10]. This combination of the Amber force field, Berger lipid parameters, and TIP3P water has been used previously [10], [36]. As an additional validation, we calculate the area per lipid head group in equilibrated POPC bilayers to be 0.69 nm^2^, closely matching the experimental value of 0.683±0.15 at 303K [37]. Data reported are for simulations with charged N- and C-termini on the peptide.

Simulations were performed using GROMACS 4.5 [38]; 200 simulations were run per mutant with an average length of 215 ns per simulation for an aggregate of 368 peptide-microseconds. Simulation details were as follows: a constant temperature of 310K (37C) was maintained using the velocity-rescaling thermostat [39], and pressure was maintained semi-isotropically at 1 bar via the Berendsen method. All covalent bond lengths were constrained using LINCS [40], and long-range electrostatics were computed every step using Particle Mesh Ewald (PME) [41].

The extensive simulations reported here were designed to probe equilibrium properties of the fusion peptide conformational ensemble. However, in examining the simulation trajectories, we detected some extremely long-lived states (decorrelation times >200 ns), such that we believe estimates of equilibrium properties may be prone to error. This finding is perhaps not surprising—for as small a protein as Trp-zip in water, prior simulation studies have found long-lived misfolded states [42], [43]. It is possible that decorrelation times in a membrane environment may be even slower. We have thus elected to report the results of a more conservative analysis; methods to analyze the equilibrium distribution of peptide conformations remain a subject of active inquiry and future work.

Unrestrained simulations were run using the Folding@Home [44] distributed computing network. In the restrained simulations, distance restraints were applied between every pair of backbone atoms within 7 Å of each other at a force constant of 1000 KJ mol^−1^ nm^−2^. The following conformational models were used as targets for the restrained simulations: the X-31 NMR structure in micelles by Han and co-workers [45] (PDB code 1IBN) for the kinked helix, the A/swine/Scotland/410440/94 NMR structure in micelles by Lorieau and co-workers [17] (PDB code 2KXA) for the helical hairpin, and the G1V X-31 NMR structure in micelles (PDB code 1XOP) by Li and co-workers [24] as an idealized straight helix. Simulations were run with and without protonation of residue Glu11; the same relationship in tail protrusion probability was observed with and without protonation of Glu11. Figures in the main text display analysis performed on the unprotonated systems, and data for protonated Glu11 is plotted in Fig. S3. All simulations are summarized in Table 1, and additional details of the analysis are given in the Text S1 as well as sequences of all simulated peptides.

**Table 1:** Summary of simulations performed.

Sequence	Structure	Simulation time
X-31	unrestrained	147 µs
G1V mutant	unrestrained	129 µs
G1S mutant	unrestrained	125 µs
X-31	Kinked helix restraints	1.5 µs
X-31	Straight helix restraints	1.5 µs
X-31	Helical hairpin restraints	1.5 µs
X-31 Glu11-H	unrestrained	1.2 µs
G1V Glu11-H	unrestrained	0.9 µs
G1S Glu11-H	unrestrained	0.9 µs

For each simulation, the starting sequence and any structural restraints are given as well as the aggregate simulation time measured in peptide-microseconds. Glu11-H denotes protonation of the glutamate 11 residue.

## Supporting Information

Figure S1 Calculated carbon-deuterium order parameters (sn1 chain only) for simulations of peptide-free POPC bilayers (blue) and ones containing X-31 influenza hemagglutinin residues 1–20 at a 1∶167 peptide-lipid ratio (red). There is a slight average shift in the order parameter induced by the fusion peptides at this peptide∶lipid ratio, but both membranes still maintain a well-behaved lamellar stucture.
(EPS)

Figure S2 Time autocorrelation functions for lipid protrusion. The autocorrelation functions show no significant difference in the average protrusion lifetime induced by different fusion peptide conformations. Upper and lower bounds represent 95% confidence intervals obtained using bootstrap resampling on the lipid protrusion time-series data. Some lipids show a tendency to flicker rapidly in and out, as can be seen from the initial period of rapid decay in the autocorrelation function. Time constants for double exponential fits are shown in Table S1.
(EPS)

Figure S3 Lipid tail protrusion for protonated Glu-11. Protrusion probability is plotted in as a function of distance from the nearest peptide. As for the non-protonated protrusion rates (Fig. 1a), the protrusion probability is greatest for fusion peptides with an N-terminal glycine (X-31 wild type), lower for the G1S mutant, and lowest for the G1V mutant. These data represent 3–4 simulations per mutant but fall within statistical uncertainty of the more extensive unprotonated simulation data.
(EPS)

Figure S4 Structural snapshot of a lipid straddling a membrane-inserted fusion peptide. Insertion of the fusion peptide into the lipid bilayer leaves a void space that must be filled above the peptide. Occasionally a lipid will occupy this space such that one acyl tail extends on either side of the peptide, thus “straddling” the peptide. Although most lipids straddling the peptide do not display acyl tail protrusion, the probability of protrusion in our simulations is significantly more likely for straddling lipids than matched lipids at the same distance from the peptide (p<0.01, Kolmogorov-Smirnov test). The straddling lipid and the fusion peptide are rendered in green sticks; other phorphorus atoms are rendered in orange spheres.
(EPS)

Figure S5 Histograms of interaction energies between peptides and lipids within 1.2 nm of each other. The top two panels show Coulomb energy for (left) protruding and (right) non-protruding lipids, and the bottom two panels show LJ-energies (protruding, left, and non-protruding, right). Although there is a slight increase in low-energy states among protruding lipids, these differences are not statistically significant (p>0.75, Kolmogorov-Smirnov test).
(EPS)

Figure S6 Protrusion probabilities in simulations of a GLIC ion channel where either the top or the bottom leaflet had 10% of its lipids removed [48]. Error bars denote 90% confidence intervals computed by bootstrap over lipids. The resulting pressure imbalance does cause a slight increase in lipid tail protrusion. However, the effect on protrusion from the X-31 fusion peptide is substantially greater, spatially localized, and results from a smaller perturbation to lipid surface area.
(EPS)

Table S1 Time constants (ns^−1^) for the autocorrelation functions in Fig. S2 after best fit to a double exponential a*exp(b*x)+c*exp(d*x) using Matlab. Upper and lower bounds represent 95% confidence intervals were obtained using bootstrap resampling.
(DOCX)

Text S1 Supporting methods.
(DOCX)
