# Supplementary material for: Lipid Tail Protrusion in Simulations Predicts Fusogenic Activity of Influenza Fusion Peptide Mutants and Conformational Models
Source: PLoS Comput Biol. 2013 Mar 7;9(3):e1002950. doi: 10.1371/journal.pcbi.1002950 (PMC3591293; doi:10.1371/journal.pcbi.1002950)
Supplement: Table S1 — Time constants (ns−1) for the autocorrelation functions in Fig. S2 after best fit to a double exponential a*exp(b*x)+c*exp(d*x) using Matlab. Upper and lower bounds represent 95% confidence intervals were obtained using bootstrap resampling. (DOCX) [file pcbi.1002950.s007.docx]

|  | **τ_fast_ (CI in parentheses)** | **τ_slow_ (CI in parentheses)** |
| --- | --- | --- |
| **Kinked helix** | 0.55 (0.49,0.62) | 0.003 (0.0025,0.0051) |
| **Straight helix** | 0.51 (0.47,0.57) | 0.007 (0.0059,0.0083) |
| **Helical hairpin** | 0.54 (0.47,0.61) | 0.008 (0.0073,0.0093) |
