# Supplementary material for: Lipid Tail Protrusion in Simulations Predicts Fusogenic Activity of Influenza Fusion Peptide Mutants and Conformational Models
Source: PLoS Comput Biol. 2013 Mar 7;9(3):e1002950. doi: 10.1371/journal.pcbi.1002950 (PMC3591293; doi:10.1371/journal.pcbi.1002950)
Supplement: Text S1 — Supporting methods. (DOCX) [file pcbi.1002950.s008.docx]

**Text S1**

Sequences of all simulated peptides were GLFGAIAGFIENGWEGMIDG (X-31), VLFGAIAGFIENGWEGMIDG (G1V), SGLFGAIAGFIENGWEGMIDG (G1S) and GLFGAIAGFIENGWEGMIDG (A/Swine/Scotland/94).

Conformational analysis was performed via complete-linkage hierarchical clustering. To make hierarchical clustering computationally feasible, we first clustered peptide conformations taken every 1 ns of simulation into 5053 clusters via k-centers clustering. Alpha-carbon RMSD was used as a distance metric. Initial k-centers clustering was performed using the MSMBuilder package[1], and subsequent hierarchical clustering was performed using Scipy, with the kink-angles as clustering coordinate. Average kink-angles in Figure 5 and 6 were calculated at a linkage distance of 10 degrees.

Kink angles were calculated using the angle formed by the alpha carbons of residues 2, 13, and 18. This simple approach resulted in more numerically stable results than fitting an ideal alpha helix to each region and calculating the angle formed between the two.

**REFERENCES:**

1. Bowman GR, Huang X, Pande VS (2009) Using generalized ensemble simulations and Markov state models to identify conformational states. Methods 49: 197–201.
